# EEG as a neural measure of hypoxia-related impairment

**DOI:** 10.3389/fcogn.2025.1503028

**Published:** 2025-02-06

**Authors:** Stephanie R. Otto, Cammi K. Borden, Daniel G. McHail, Kara J. Blacker

**Affiliations:** ^1^Naval Medical Research Unit-Dayton, Wright-Patterson AFB, Dayton, OH, United States; ^2^Applied Cognitive Neuroscience, Oak Ridge Institute of Science and Education (ORISE), Oak Ridge, TN, United States; ^3^Leidos Inc., Reston, VA, United States

**Keywords:** hypoxia, EEG, event-related potentials, military aviation, physiological monitoring

## Abstract

Ambient oxygen decreases with increasing altitude, which poses a primary threat to aviators known as hypoxic hypoxia. Decades of research have shown that hypoxia impairs cognition, but the neurophysiological bases for these effects remain poorly understood. Recent advances in neuroscience have permitted non-invasive observation of neural activity under controlled hypoxia exposures and have begun to uncover how the brain responds to hypoxia. Electroencephalography (EEG) in particular has been used to explore how electrical activity produced by networks of cortical neurons changes under hypoxia. Here we review studies that have explored how hypoxia affects prominent EEG brain rhythms as well as responses to specific events or stimuli in the time and frequency domains. Experimental conditions have varied widely, including whether hypoxia exposures were normobaric or hypobaric and the range of equivalent altitudes and durations of exposures. Collectively, these studies have accumulated support for a variety of candidate neural markers of hypoxia impairment spanning sensory and cognitive domains. Continued research will build on these findings to leverage emerging technologies in neuroscience and further our understanding of how hypoxia affects cognition and associated neural activity.

## 1 Introduction

Environmental oxygen decreases with altitude, which limits the amount of oxygen available to the brain. This poses a hazard to aviators known as hypoxic hypoxia. It is well known that hypoxic exposure negatively impacts cognition and can impair a pilot's performance and increase the risk of a mishap. Studies have begun to differentiate how hypoxia impairs different aspects of cognition and, more recently, associated brain function. In this review we highlight progress in this field and where additional research is needed to further our understanding of the hypoxic brain.

Tactical aviators are exposed to physiological stressors every time they fly due to the nature of the high-performance jet cockpit, putting them at risk of experiencing unexplained physiological episodes (UPE) and degraded performance. A UPE is defined as “when aircrew experience adverse physiological, psychological, pathological, or physical problems that manifest during or after flight” (DeNicola and Corner, 2023). Between 2007 and 2019, the F-22, F-35, F/A-18, F-15, F-16, T-6, and T-45 have each reported at least 10 UPEs that are not explained by classic physiological training. The F/A-18, operated both on and off aircraft carriers by the US and foreign governments, has reported more than 603 UPEs in the same period of time (Elliott and Schmitt, 2019). While there are a number of various putative causes to UPEs, one that received the most attention early on was hypoxia. Although aircraft life support systems and hypoxia familiarization training are effective countermeasures for hypoxia, it remains a leading threat in aviation. There is currently no sensor in the cockpit that can reliably detect impairment due to hypoxia. The primary alerting system for hypoxia remains the aviator, who is trained to recognize the symptoms of hypoxia and subsequently execute emergency maneuvers. However, hypoxia can compromise an individual's ability to realize that they are impaired, and self-reported symptoms of hypoxia may vary widely both between and within individuals (Cox et al., 2024). Therefore, physiological and neural indicators of pilot state are needed as training adjuncts and for incorporation into in-cockpit alerting and auto-recovery systems. Understanding the neural bases of hypoxia can help improve these countermeasures and enhance aviator performance and survivability.

Different approaches to modeling hypoxia exposure and recording neural activity in a research setting have begun to uncover the complexity of neural responses to hypoxia. One growing approach to measuring hypoxia related impairment is electroencephalography (EEG). EEG is a non-invasive measure of the electrical activity in the brain that is measured via electrodes placed on the scalp. EEG is a direct measure of neural activity at the level of populations of neurons and has millisecond level precision. There are three major classes of data that can be leveraged from EEG, which we will discuss in this review paper. First, we can examine spectral power fluctuations in a number of traditionally agreed upon frequency bands: delta (1–3 Hz), theta (4–7 Hz), alpha (8–13 Hz), and beta (14–30 Hz). These frequency bands reflect the coordinated activity of populations of neurons and studying different frequency bands in different cortical areas can reveal impacts of hypoxia on different aspects of cognition. While informative, these measures evaluate spectral activity across wide windows of time and are less sensitive to the transient shifts in neural activity that EEG can also capture. Essentially, spectral analysis of large amounts of time negates the advantage that EEG provides with good temporal resolution. Therefore, a second approach to using EEG is to examine event-related potential (ERP) components. ERPs are stimulus evoked responses that occur on a millisecond timescale and provide a direct measure of brain activity associated with sensory, cognitive, and motor events. Many ERP components have decades of research to support their time course, neural generators, and functional associations (Luck, 2014; Luck and Kappenman, 2013). Finally, an increasingly popular approach to EEG is examining event-related fluctuations in rhythmic oscillatory EEG activity. This approach is often referred to as time-frequency analysis and allows examination of time-varying power in different wavebands time-locked to stimuli. Time-frequency content includes phase-locked activity (largely forming ERPs in the time domain) as well as non-phase-locked activity (for a more in-depth explanation, see Bastiaansen et al., 2013). In sum, event-related oscillatory responses are modulations of ongoing activity.

In addition to these three classes of data that can be utilized for EEG and hypoxia research, there are also advancements occurring that provide additional measures such as functional connectivity (Schoffelen and Gross, 2009), non-linear dynamics (Stam, 2005), and machine learning (Hosseini et al., 2020). However, these measures are beyond the scope of the current review because to date, few studies have employed these measures to study acute hypoxia. Moreover, there have been hardware related advancements for EEG that may bolster future efforts for in-cockpit monitoring such as dry EEG (e.g., Kam et al., 2019), in-ear EEG (e.g., Mikkelsen et al., 2015), or helmet integrated EEG. Together, these technological advances make the possibility of using EEG in an aircraft environment plausible with future development. Here, we review the extant literature on spectral power, ERPs, and event-related oscillatory changes during acute hypoxia exposure.

## 2 Method

PubMed, ProQuest, EBSCO Discovery Service, and Google Scholar databases were used to search for peer-reviewed journal articles. A manual search was performed by a combination of the following key terms: acute hypoxia, hypoxic hypoxia, normobaric hypoxia, hypobaric hypoxia, EEG, spectral analysis, ERP, and time frequency. Articles were then independently screened based on a set eligibility criterion. First, articles had to be published in peer-reviewed journals. Second, articles were original research. Third, research was on human participants rather than animals. Fourth, the studies examined hypoxic hypoxia, other forms of hypoxia such as ischemic or anemic hypoxia were not included. Finally, the study must have included EEG recording. We included both normobaric and hypobaric hypoxia exposures.

## 3 Effects of hypoxia on human electrophysiology

### 3.1 Changes in EEG spectral activity associated with hypoxia

Oscillations in EEG activity reflect the coordinated activity of populations of neurons underlying different aspects of neurocognitive function, and experiments have begun to uncover effects of hypoxia on these brain rhythms. Experimental approaches have varied widely, including the type of exposure (normobaric or hypobaric), exposure altitude and duration, and whether EEG was recorded during either eyes-open or eyes-closed resting state or performance of a cognitive task. Collectively, the literature covers impacts of hypoxia on four discrete sub-bands (delta, theta, alpha, and beta in order of increasing frequency). Experimental conditions and findings of these studies are summarized in Table 1.

**Table 1:** Hypoxia and spectral analysis studies reviewed.

**References**	**Altitude (O_2_ %) Exposure minutes**	**Eyes opened/closed**	**Method of EEG analysis**	**Synopsis of hypoxia effects**
Kraaier et al. (1988)	Hypobaric 20k ft (9.7%) 17 min	Eyes-closed resting state	Power spectral density, subtraction spectra between conditions	Decrease in alpha power and frequency Increase in delta and theta power, no change in beta
Van der Worp et al. (1991)	Normobaric SpO_2_ adjustable Until SpO_2_ threshold met	Eyes-closed resting state	Subtraction spectra	At 60% SpO_2_: decrease in alpha power and frequency, increase in lower frequencies (including delta and theta)
Ozaki et al. (1995)	Hypobaric Gradual 3k m, 4k m, 5k m, 6k m (9.7%−14.3%) Each 25 min	Eyes-closed resting state	FFT and subtraction spectra	Decreased alpha power, esp. posterior Increased anterior theta power Delayed recovery
Schellart and Reits (2001)	Normobaric Calculated altitude of ~12k ft−29k ft (7%−13%; exposure generally was 10%) 30–60 min	Alternating eyes-open and eyes-closed throughout exposure	FFT for consecutive windows < 1 min throughout the exposure	Beta, Theta, and Delta power increased for eyes-open and eyes-closed activity over occipital cortex Eyes-closed alpha power decreased. Smaller eyes-closed/eyes-open ratio with hypoxia. Eyes-open and eyes-closed alpha frequency decreased
Papadelis et al. (2007)	25k, 20k, and 15k ft (11.8%−8.1%) 3 min per altitude	Eyes-open, performing cognitive task	FFT for 5 s epochs, averaged ApEn	Marginally increased delta, increased theta and alpha power. Lower ApEn
Schneider and Struder (2009)	Normobaric 12.7 kPa PIO_2_ for 40 min	Eyes-open, performing cognitive task	sLORETA	While performing a pencil-tracking task, increased beta over right superior frontal gyrus, no change in alpha
Hutcheon et al. (2023a)	5 min exposures, either hypobaric at 3,962 m (12.7%) or normobaric at 12.8% O_2_	Eyes-open resting state	Absolute and relative power computed with Welch's method	Alpha and beta power negatively correlated with SpO_2_, delta and theta positively correlated
Beer et al. (2024)	Hypobaric 25k ft (8.1% O_2_; separate sessions to ascend rapidly and gradually) 10 min or until other safety criteria were met	Eyes-opened	RMANOVA performed on four separate epochs of each frequency band	Both the Delta and Beta showed a significant increase during the hypoxic exposure vs. the 100% O_2_ exposure from both the gradual and rapid onset

Altitude to O_2_ comparisons are from the Hypoxico Altitude to Oxygen Chart (Hypoxico Altitude Training Systems, n.d.).

FFT, Fast Fourier Transform; ApEn, Approximate entropy; kPa, kilopascal; PIO_2_, Partial pressure of inspired oxygen; sLORETA, standardized low-resolution brain electromagnetic tomography; RMANOVA, Repeated measures analysis of variance.

The delta rhythm is typically elevated only during sleep, anesthesia, or epileptogenic activity. Hypoxia has been associated with increased delta activity during the eyes-closed resting state under both hypobaric (Kraaier et al., 1988) and normobaric (Van der Worp et al., 1991) exposures. Eyes-open delta during resting state similarly increased with hypoxia (Schellart and Reits, 2001). While engaged in a cognitive task, there is also evidence for increased delta power during hypoxia compared to normoxia (Papadelis et al., 2007; Beer et al., 2024).

Theta oscillations coordinate neural networks involved in memory and other cognitive processes. Similarly to the delta sub-band, hypoxia is associated with increased theta activity during the resting state (Kraaier et al., 1988; Van der Worp et al., 1991; Schellart and Reits, 2001, although see Hutcheon et al., 2023a for a contrary finding). In particular, Ozaki et al. (1995) found that increases in theta under hypoxia were most pronounced over frontal cortex during the resting state. Theta was also elevated during performance of a computer-based flight simulation task (Papadelis et al., 2007).

The alpha rhythm tends to dominate during relaxed wakefulness when the eyes are closed but decreases during engagement with a task or when the eyes are open. Compared with normoxia, alpha power and frequency decrease during eyes-closed resting state EEG, both under hypobaric (Kraaier et al., 1988) and normobaric (Van der Worp et al., 1991) hypoxic exposures. During an eyes-closed resting exposure, Ozaki et al. (1995) found these effects were widespread in the cortex but particularly pronounced over occipital regions. In contrast to eyes-closed alpha, eyes-open alpha increased under hypoxia (Schellart and Reits, 2001; Hutcheon et al., 2023a). The ratio of eyes-closed to eyes-open alpha power was also reduced (Schellart and Reits, 2001). When a computer-based flight simulation task was performed under hypoxia, alpha power also increased, although these effects were limited to more extreme altitudes (Papadelis et al., 2007). In addition, some studies have examined the “hypoxia hangover” effect and found delayed recovery of alpha to baseline levels following the exposure (Ozaki et al., 1995), while under different exposure and experimental conditions recovery is immediate (Schellart and Reits, 2001). In summary, a range of experimental conditions have demonstrated the sensitivity of alpha to hypoxia, but the direction and duration of these impacts depend on factors including exposure severity and the task performed during the exposure.

Beta activity is most often studied over central sites due to its role in motor planning and execution, but in the absence of a task it is associated with alertness. Beta was not affected by hypoxia during an eyes-closed hypobaric exposure (Kraaier et al., 1988), although a non-significant trend for increased beta was present. However, during eyes-open resting exposures, hypoxia was significantly associated with increased beta (Schellart and Reits, 2001; Hutcheon et al., 2023a). Additionally, beta activity has been shown to increase during a visuomotor task across the central electrodes during hypobaric hypoxia (Beer et al., 2024) and localized to the right superior frontal gyrus (Schneider and Struder, 2009).

In summary, under a variety of exposure conditions the literature shows consensus that hypoxia is associated with increased delta, theta, and beta power during resting-state EEG. Alpha power also increases with hypoxia during eyes-open resting state EEG but decreases when the eyes are closed. Spectral changes related to hypoxia during task performance are relatively underreported but may index deficits in cognitive function associated with hypoxia.

Of note, many of the studies listed in this section reported high levels of interindividual variability in spectral responses to hypoxia. Future studies should further explore underlying factors for these individual differences, including those that contribute to a possible trait of hypoxia vulnerability or resilience. In addition, a few studies that included repeated exposures found that EEG responses varied within individuals. This suggests that unknown, state-based factors may also contribute to responses of spectral activity to hypoxia, and these should be further investigated.

### 3.2 ERPs and hypoxia

Early studies that used spectral analysis to investigate the effects of hypoxia on neural activity provided preliminary evidence demonstrating the potential consequences of hypoxic exposure on neurocognitive functioning. However, these studies lacked the temporal precision required to understand at what level of neural processing are sensory or cognitive functions impaired. This limitation has made it difficult to extrapolate findings to specific sensory or cognitive processes and ERPs have emerged as a promising solution to this problem.

An ERP is a measured brain response that is a direct reflection of a specific sensory, cognitive, or motor event. Researchers discovered that neural signals could be indexed, or time-locked, to a specific stimulus event. This produces an ERP waveform that represents the brain's response to a particular stimulus with millisecond precision. ERPs have made it possible to determine which stage of information processing is being affected under various health and diseased states. Thus, event-related experimental research designs, together with ERP analysis methods, have emerged as a temporally superior methodology in determining the degree to which cerebral functioning is perturbed across a multitude of sensory and higher-order cognitive domains.

The ERP waveform can be decomposed according to a series of negative and positive going deflections following the stimulus presentation. Taken together, these deflections are often referred to as ERP components and they can inform researchers about where in the processing of information within the brain does something like acute hypoxia have an effect. Broadly speaking, ERPs are often classified as “cognitive” or “sensory” ERPs. Later ERPs in ~200–400 ms range reflect cognitive processing of external stimuli and tend to involve attention, working memory, decision-making, etc. This cognitive processing of sensory stimuli is preceded by stimulus transduction in sensory organs and conduction of neural signals along the sensory pathways (Pratt, 2011). The recording and analysis of sensory ERPs have been widely implemented across research and clinical practices due to their sensitivity in measuring the integrity of the sensory input to more complex central nervous system processes (Luck and Kappenman, 2013). A summary of the hypoxia and ERP literature discussed below can be found in Table 2.

**Table 2:** Hypoxia and ERPs research studies reviewed.

**References**	**Altitude (O_2_ %) Exposure minutes**	**Eyes opened/ closed**	**ERP(s) of interest**	**Method of EEG analysis**	**Synopsis of hypoxia effects**
Kida and Imai (1993)	Hypobaric 3k, 4k, 5k, and 6k m (~9.7%−14.3%) ~45 min each	Eyes closed	NSW PSW N1 N2 P2 P3	Changes in mean amplitudes and mean latencies. Grand averaging	Participants that didn't show any significant changes to their RT showed no changes in the ERP components. Participants that showed significant changes to their RT showed delayed peak latencies and impaired amplitudes
Lucertini et al. (1993)	Hypobaric 17k ft (11%) for 30 min. Additional 7 min to ascend and descend	Not specified	SSRs (P1, N1) MLRs (Pa, Na)	Changes in mean amplitudes and mean latencies	Found no significant changes in the MLR waveforms but did see significant differences with the SSRs
Urbani and Lucertini (1994)	Hypobaric 17k ft (11%) for 90 min. Additional 7 min to ascend and descend	Not specified	ABR waves I, III, and V	Changes in mean amplitudes and mean latencies	Impaired latencies across all waves, at either 90 min in or during recovery
Bouchet et al. (1997)	Hypobaric 4,350 m (~12%) for 5–6 days. Recordings at 24 h and 72 h	Eyes opened	MLAEPs (V, Na, Pa, Nb, and Pb)	Changes in mean amplitudes and mean latencies	No significant changes or influence of hypoxia was observed on latency or amplitude of the MLAEPs
Singh et al. (2004)	Hypobaric 3,200 m (~14%) for 6 days and then 4,300 m (~12.3%) for 8 days Recordings were from 0800 h−1,300 h	Eyes opened	N1 N2 P2 P3	Changes in mean amplitudes and mean latencies	P3 latency increased in more participants during the 4,300 m exposure, with the 3,200 m showing an increase in latency but in less participants
Hayashi et al. (2005)	Hypobaric Rapid ascent from 610 m to 4,500 m (~15%). 3 h exposure, including ramp to 4,500 m	Not specified	N1 P3	Changes in mean amplitudes and mean latencies	The latency of P3 was significantly increased after 2 h, and then returned to baseline following oxygen recovery. No significant changes to N1 amplitude or latency
Tsarouchas et al. (2008)	Hypobaric 15k ft (11.8%) Maximum 15 min	Eyes opened	N1 P1 N2 P2 P3	Changes in mean/peak amplitudes and mean/peak latencies. Grand averaging	P1 peak amplitude was attenuated, whereas N1 and P3 peak amplitude enhanced. Additionally, the P2 peak latency was delayed, whereas P3 peak latency increased
Nakata et al. (2017)	Normobaric Equivalent of 12% O_2_ or roughly 14.5k ft 30 min	Eyes opened	N140 P300 N100 P200	Changes in peak amplitudes and peak latencies. Grand averaging	Peak latency of P300 was significantly later under hypoxic conditions for the Go and No-Go tasks
Altbacker et al. (2019)	Normobaric SpO_2_ adjustable Until SpO_2_ threshold was met (80%)	Eyes opened	Target P3 NoGo P3 Novelty P3	Difference waves. Changes in peak amplitudes and peak latencies. Grand averaging	Significant change in the Novelty P3 amplitude from hypoxia. Additionally, there was a significant interaction from the effect of hypoxia on the Novelty P3 between Fz and Cz channels
Seech et al. (2020)	Normobaric 17.5k ft (10.6%) 27 min	Eyes opened	MMN P3a RON	Changes in mean amplitudes and mean latencies. Grand averaging	Significant reduction in P3a amplitude during hypoxia compared to normoxia
Blacker and McHail (2021)	Normobaric 20kft (9.7%) 10 min	Eyes opened	MMN P3a	Difference waves (deviant – standard) Changes in mean amplitudes and mean latencies. Grand averaging	Reduction in the MMN amplitude from hypoxia. Recovery returned to baseline levels ~2 h post exposure
Blacker et al. (2021)	Normobaric 17.5k ft (10.6%) 27 min	Eyes opened	vMMN	Difference waves (deviant – standard) Changes in mean amplitudes and mean latencies	Reduction in the vMMN amplitude during hypoxia compared to normoxia
Blacker and McHail (2022)	Normobaric 20k ft (9.7%) 14.5 min	Eyes opened	P100 P50 N100 P200	Changes in mean amplitudes and mean latencies. Grand averaging	P200 was significantly reduced during hypoxia compared to normoxia
Zani et al. (2023)	Normobaric 4,200 m (12.5%) 4 h	Eyes opened	ADAN/CNV LDAP/TP	sLORETA	Increased amplitude of ADAN and LDAP in the spatial orienting conditions for the upper visual. The lower hemifield showed increased ADAN/CNV, but decreased LDAP/TP
Borden et al. (2024)	Normobaric Gradual incline to 10k, 15k, 20k, and 25k ft (8.1%−14.3%) at 5 min intervals 30 min including baseline	Eyes opened	MMN P3a	Difference waves. Changes in mean amplitude and mean latencies	Marginally significant decrease in the P3a amplitude from normoxia to hypoxia

Altitude to O_2_ comparisons are from the Hypoxico Altitude to Oxygen Chart (Hypoxico Altitude Training Systems, n.d.).

NSW, negative slow wave; PSW, positive slow wave; RT, reaction time; SSR, steady state response; MLR, middle latency response; ABR, auditory brainstem response; MLAEP, middle latency auditory evoked potential; MMN, mismatch negativity; RON, reorienting negativity; vMMN, visual mismatch negativity; ADAN, Anterior directing attention negativity; CNV, Contingent Negative Variation; LDAP, late directing attention potential; TP, tonic positivity.

#### 3.2.1 Effects of acute hypoxia on sensory ERPs

Sensory ERPs can be broadly categorized into three domains: auditory evoked potentials, visual evoked potentials, and somatosensory evoked potentials. Auditory evoked potentials can be further classified into three subcategories according to the timing, or latency, of the elicited neural response and where along the auditory pathway a response is evoked. The earliest of these ERPs is the auditory brainstem response (ABR), in the first 10 ms after stimulus onset. Following the ABR is the middle-latency evoked responses, which are observed from ~10 ms to 60 ms post stimulus onset and finally, long-latency components are observed from 60 ms to 200 ms after stimulus onset.

The effects of hypoxia on early auditory processing, including the ABR, middle-, and long-latency components, have been evaluated using EEG under a variety of hypoxic conditions. Studies that have assessed the effects of hypobaric hypoxia on ABRs have found impairments at both 17,000 ft (Urbani and Lucertini, 1994) and at 14,750 ft equivalent altitudes (Bouchet et al., 1997; Hayashi et al., 2005). In contrast, middle-latency components do not appear to be impaired by hypoxia. An early study by Lucertini et al. (1993) examined the effects of hypobaric hypoxia during a 17,000 ft simulated exposure on both middle-latency and steady-state auditory evoked responses. They found no statistically significant effects on middle-latency components but reported a significant increase in steady-state response latencies. Similarly, Bouchet et al. (1997) found no significant differences in middle-latency components during a hypobaric hypoxia exposure at 14,250 ft altitude. Finally, no statistically significant effects of hypobaric hypoxia have been reported for long-latency response components (Hayashi et al., 2005); however, under normobaric hypoxia, Blacker and McHail (2022) found that the P200 sensory gating ratio was attenuated when compared to normoxic conditions. Their results suggest there is a disruption of the auditory system that is specific to the level of allocating attention following basic auditory processing.

The visual system is particularly sensitive to its oxygen supply at multiple levels including the retina, photoreceptors, and cortical and subcortical pathways. The adverse effects of hypoxia exposure on the physiology of the visual system have been well documented and findings suggest that hypoxia may have serious consequences for a multitude of human performance outcomes. The two primary cell types of the retina require enormous metabolic demands both in darkness and light, and processing visual information requires a vast and continuous supply of energy and oxygen.

Studies investigating the unique effects of hypoxia on early visual processing have found that early sensory ERP components, mainly the P1 and P2 amplitudes, are attenuated during a 15,000 ft hypobaric hypoxia exposure and a significant delay of the P2 peak latency was also observed (Tsarouchas et al., 2008). In contrast, a study by Blacker and McHail (2022) investigated the effects of normobaric hypoxia on early visual evoked potentials using a pattern reversal-paradigm at 20,000 ft simulated altitude. Initial results indicated that there was a significant reduction in the amplitude of the P100 during the experimental condition, however, upon further investigation and high pass filtering of the ERP data, those results no longer reached significance.

Despite these contrasting findings, there is an established literature demonstrating that functions of the primary visual system (visual acuity, luminance, etc.) are one of the first sensory systems to be affected by hypoxia, and this likely has downstream effects on cognitive functions. The later ERP components reflecting the higher order cognitive effects of hypoxia on auditory and visual processing are discussed below.

#### 3.2.2 Effects of acute hypoxia on cognitive ERPs

One of the most thoroughly studied ERP paradigms with respect to acute hypoxia is the oddball paradigm. In an oddball paradigm, a standard stimulus is presented the majority of the time and occasionally an oddball or deviant stimulus is presented. Oddball stimuli can be from a number of sensory domains including auditory, visual, somatosensory, etc. For example, in a classic auditory oddball paradigm, a standard tone (e.g., 50 ms, 1,000 Hz) is presented ~85% of the time and randomly interspersed are oddball tones (e.g., 100 ms, 1,000 Hz) the other 15% of time. This type of paradigm can either be active or passive. In an active oddball, participants are asked to track the stimuli and respond in some way; whereas, in a passive paradigm, participants complete some other central task and are instructed to ignore the oddball stimuli.

When using an active auditory oddball paradigm under hypoxia, Altbacker et al. (2019) examined three variants of the P300 component and found that the “Novelty P3,” which was elicited by a task-irrelevant novel stimulus, was decreased in amplitude during normobaric hypoxia compared to normoxia. The authors concluded that hypoxia resulted in an impairment to novelty processing, while leaving task-relevant information processing unaffected. An earlier study by Kida and Imai (1993) investigated the effects of hypobaric hypoxia on N2 and P3 ERP components while using an active auditory oddball paradigm where participants were asked to discriminate between two different pip tones. They found significant changes in latency and amplitude for both the N2 and P3 components which were thought to reflect sensory discrimination and processing. Finally, a study by Singh et al. (2004) investigated the effects of hypobaric hypoxia during two separate high-altitude conditions (10,500 ft and 14,100 ft) while using an active auditory oddball paradigm. Their results showed a significant increase in the latency of the P3 component, and this effect continued to deteriorate as altitude increased.

Several prior studies have examined the effects of acute normobaric hypoxia on the mismatch negativity (MMN) and P3a components that are elicited from a passive oddball paradigm. The MMN/P3a complex is thought to reliably index automatic and pre-attentive stages of early information processing and were first measured using auditory stimuli (e.g., Naatanen et al., 1978, 2007, 2019). The first study to investigate the MMN/P3a under acute hypoxia conditions, found a significant reduction in the amplitude of the P3a during a 27-min 17,500 ft equivalent normobaric exposure, compared to a normoxia control condition (Seech et al., 2020). Next, a replication in the visual domain was conducted with the same parameters, except a passive visual oddball paradigm was used and similarly Blacker et al. (2021) found a reduction in the visual MMN during hypoxia compared to normoxia.

Moreover, it has been demonstrated that not only is there a reduction in amplitude of these components during acute hypoxia, but that this reduction persists for up to 2 h post-exposure, suggesting a lag in neurocognitive recovery (Blacker and McHail, 2021). Finally, in the most recent study in this series, the time course of neurocognitive impairment during a gradual normobaric, mask-on, exposure was examined. The work demonstrated that individuals going through an exposure profile, similar to a U.S. Navy hypoxia familiarization protocol, had a decreased P3a amplitude and the timing of that reduction was on average about as quick as participants could subjectively identify and report their symptoms (Borden et al., 2024).

Finally, a handful of other studies have examined other ERP components. A study by Zani et al. (2023) examined the effects of hypoxia on visuospatial attention using a modified Attention Network Test (ANT) during either a normoxic control condition or a normobaric hypoxia condition at a simulated altitude of 13,780 ft. Their results indicated that ERPs to pre-target cues elicited both an Anterior directing attention negativity (ADAN)/CNV and a posterior Late directing attention positivity (LDAP)/TP, which in ambient air were larger for attention orienting than for alerting. The hypoxia condition resulted in increased amplitude of both these potentials in the spatial orienting conditions for the upper visual hemifield, while, for the lower hemifield, it increased ADAN/CNV, but decreased LDAP/TP for the same attention conditions. Moreover, a study by Nakata et al. (2017) investigated the effects of normobaric hypoxia on somatosensory evoked potentials (SEPs), motor execution and inhibitory processing ERPs using a Go/No-go paradigm at a simulated altitude of 14,000 ft. Compared to normoxia, the high-altitude condition resulted in a significant reduction of the peak amplitudes of the Go-P300 and No-go-P300, as well as a significant delay in the latency of the Go-P300. However, they did not report any significant findings related to the SEPs. These results suggest that acute hypoxia exposure impaired executive functioning for motor and inhibitory processes and demonstrated a significant lag in latency for motor executive processes.

### 3.3 Time-frequency and hypoxia

Combining strengths of spectral analysis and ERPs, a third approach assesses changes in brain oscillations in response to specific events. Termed time-frequency analysis, this method leverages the temporal specificity of EEG by observing spectral activity in discrete time windows following experimental stimuli. A limited number of studies have used time-frequency analysis to investigate neural responses to hypoxia.

Zani et al. (2020) used time-frequency analysis to investigate changes in visuospatial attention during a normobaric hypoxia (13,780 ft equivalent altitude) exposure. They found that hypoxia was associated with impaired visuospatial attention as well as increased alpha power immediately following task stimuli, specifically over the right parieto-occipital cortex, the medial-occipital cortex bilaterally, and left dorsolateral prefrontal cortex. This selective increase of alpha power under hypoxia may underly compensatory activity by the brain to focus attention toward relevant visual stimuli. Thus, by time-locking their analysis to a visuospatial attention task, the researchers extended previous work by tying hypoxia impairments to specific cognitive deficits and changes in the activity of associated brain regions.

Building on work by Zani et al. (2020), Hutcheon et al. (2023b) also observed changes in oscillatory activities time-locked to stimuli in a visuospatial attention task during hypoxia, but used a normobaric (12.8% oxygen) as well as a hypobaric (13,000 ft equivalent) hypoxia exposure. They computed evoked power (phase-locked) as well as induced power (not phase-locked) in different frequency bands following event onset. They found that, in the first 300 ms following stimuli, under normoxia induced alpha power increased over parietal cortex, while under hypoxia (both normobaric and hypobaric) alpha was instead elevated over left prefrontal cortex. This finding is similar to that by Zani et al. (2020) and could reflect compensatory interregional shifting of brain resources to maintain visuospatial attention in response to hypoxia. Alpha dynamics shifted after 300 ms following stimuli, such that induced alpha power over parietal and central cortex decreased under hypoxia compared with normoxia. Taken together with decreases in task performance under hypoxia, this decrease in alpha could reflect hypoxia-related impairment of neural systems supporting attentional control. In contrast to induced alpha, induced lower-beta power was elevated under hypobaric but not normobaric normoxia. Relative to stimulus onset, this increase in lower-beta power began in left frontal-parietal cortex and progressed posteriorly to parietal cortex. Evoked higher-beta power was also elevated under hypobaric hypoxia compared with normobaric hypoxia and normoxia, particularly over frontal regions. The authors suggest that the perturbation of beta specific to hypobaric hypoxia remains open to interpretation but may be connected to changes in breathing dynamics connected with increased top-down attentional control. That inclusion of hypobaric as well as normobaric hypoxia conditions uncovered effects that differed by oscillatory bands suggests that future studies of how hypoxia impacts brain rhythms should also consider potential differences between hypobaric and normobaric exposures. Future studies should also take note that, as illustrated by the differing activity in alpha in the time following stimulus presentation, responses of oscillatory activities to hypoxia may be transient and require analytical approaches with adequate spatiotemporal sensitivity to capture.

While valuable for understanding impacts of hypoxia on neural oscillatory activities in the laboratory, time-frequency analysis has important limitations for real-time detection of hypoxia impairment in an operational setting. In particular, the stimuli required for time-frequency analysis may distract pilots during flight. Also, the large number of trials required for time-frequency analysis surpasses the number required for ERP analysis, further limiting feasibility of its real-time applicability in the cockpit.

Finally, we note that additional information is available in EEG beyond time-frequency analysis and the other methods previously discussed. Like other complex natural systems, changes in brain activity over time can be modeled using mathematical tools from the field of non-linear dynamics (Stam, 2005). One of the first teams to apply non-linear dynamic analysis to EEG data under hypoxia, Papadelis et al. (2007) computed the approximate entropy (ApEn) of the EEG during hypoxia. They found that ApEn, a measure of complexity, decreased with hypoxia, particularly at higher altitudes. Interpretations of changes in signal complexity with regard to brain activity remain open to discussion but could reflect reduced communication between brain regions. Future experiments that directly examine neural connectivity under hypoxia are encouraged, as are those that use non-linear dynamic analysis to model changes in brain activity under hypoxia.

## 4 Why use EEG to study hypoxia?

One common question in this line of research is why should we use EEG to study the effects of hypoxia at all? Traditional approaches to assessing hypoxia in the context of military aviation or mountaineering focus on peripheral oxygen saturation (SpO_2_) and subjective symptom reporting. For example, the U.S. Navy and Air Force have been using hypoxia familiarization training since the 1940s, which serves to expose aircrew to a controlled hypoxic exposure so that they can identify their symptoms. While this is a useful tool for teaching aircrew what a hypoxic exposure is like, there are inherent problems with self-reported symptomology. Cox et al. (2024) recently demonstrated at least half of individuals report symptoms *inconsistently* across repeated exposures separated by a matter of days. Secondly, while SpO_2_ is an objective measure of oxygen content in the periphery and can determine whether someone is physiologically hypoxic, it has nearly no predictive power for determining performance and/or impairment during a hypoxic exposure. For example, in one recent study by our group, we demonstrated that SpO_2_ and heart rate recover back to baseline levels within a few minutes of breathing air or 100% oxygen, but reaction time and ERP amplitude did not return to normal for 60 min and 120 min, respectively (Blacker and McHail, 2021). This suggests that peripheral physiological measures are not sensitive to neurocognitive dysfunction that can and does occur under hypoxic conditions and thereafter. Importantly for in-cockpit applications, EEG measures can index cognitive function passively, without imposing additional workload or distractors on pilots. Future research should also assess whether changes in EEG activity might predict performance deficits due to hypoxia. Earlier detection of impending hypoxia impairment, potentially using EEG, would be a valuable addition to hypoxia familiarization training and to early warning systems during flight.

## 5 Discussion

Low oxygen exposure inevitably occurs with ascent to altitude and can occur during aviation or mountaineering. A reduction in breathable oxygen results in hypoxic hypoxia and has been a concern in military tactical aviation for some time now. While cockpits are pressurized and aircrew breathe from a life support system, issues can arise that may result in a hypoxic exposure, which poses grave risk to the aircrew and aircraft. There are two major gaps in knowledge surrounding acute hypoxia exposure. First, there are wide ranging individual differences in both physiological (e.g., SpO_2_ and heart rate) and performance (e.g., reaction time, aircraft control) outcomes. Moreover, there is not a strong relationship between those physiological and performance outcomes, which makes simply monitoring SpO_2_ ineffective at assessing who will experience or when someone will experience a performance deficit. Second, it remains unclear what neurophysiological mechanisms underlie hypoxia-related cognitive impairment. As early as the 1980s, researchers have been quantitatively using EEG to assess neurocognitive effects of acute hypoxia exposure. In the past decade there has been a surge in these efforts and enough evidence has accumulated to warrant this review of the literature to assess the state of the science.

The earliest approach to studying acute hypoxia with EEG was spectral power analysis. Across multiple studies, hypoxia appears to result in an increase in delta power compared to a normoxia control. Broadly speaking, our most thorough understanding of the role of delta bands comes from sleep research. During sleep, delta oscillations between the thalamus and neocortex constrain what information from the outside world is detected and passed through the thalamus to higher-order cognitive brain regions (Buzsaki, 2006). Therefore, we interpret increased delta power during acute hypoxia as indicating an altered state of consciousness, which is what we see during deep sleep and even g-induced loss of consciousness (Wilson et al., 2005). The literature also shows consistent increases in frontal theta power during acute hypoxia both during rest and while on task. Interestingly, the alpha band shows the most heterogenous results with some studies showing increased alpha and other decreased alpha. A key component of these seemingly contradictory results is whether EEG was measured during eyes closed, eyes open rest, or while performing a task. While alpha is the dominant rhythm in the brain and is associated with drowsiness and Stage 1 sleep, it also has a number of functional roles when individuals are engaged in a task, such as inhibition of task-irrelevant information (Jensen and Mazaheri, 2010). Finally, few studies have examined beta oscillations with some finding increased beta power; again, major roadblocks to drawing strong conclusions are due to the use of rest vs. on task conditions. Typically, beta over motor cortex is associated with motor planning and execution, but only one prior study examined beta during a motor task during hypoxia (Schneider and Struder, 2009).

The reviewed literature on sensory ERPs and hypoxia is difficult to interpret due to some inconsistent findings. Within the auditory domain, ABRs and long-latency potentials have shown disruption with acute hypoxia exposure, but no such findings have emerged for middle-latency potentials. It is unclear why early and late stages of auditory processing would be impaired but middle stages would be spared. Further work is needed to examine these effects. While there is a robust literature on the effects of hypoxia on the visual system, the visual ERP findings are also a bit inconsistent. One major determining factor seems to be what type of visual stimuli are used, specifically what colors are used and what type of lighting environment is present (Barbur and Connolly, 2011).

Unlike sensory ERPs, the effects of acute hypoxia on attention and cognitive ERPs have been shown to be quite consistent and reliable. A number of studies now have demonstrated that the family of P300 components is sensitive to hypoxic exposure of varying altitudes. This includes both passive paradigms examining the P3a, as well as active target and go/no-go P300 paradigms. The P300 has much functional significance with cognitive performance, as individual differences in this component are associated with mental speed and how rapidly one can allocate attentional resources (Pelosi et al., 1992; Houlihan et al., 1998). With respect to assessing overall cognitive performance in the face of many environmental stressors that are relevant to military aircrew, the P300 holds much promise.

As stated above, one of the knowledge gaps in the acute hypoxia literature is the uncertainty about the neural mechanisms underlying hypoxia's effects on performance. A promising approach to addressing this knowledge gap, is time-frequency design and analysis of EEG data. Two recent papers have shown that acute hypoxia, both hypobaric and normobaric, result in increased alpha oscillations during a visuospatial attention task and that these changes in oscillatory changes are specific to brain regions known to play a role in attention (i.e., parietal and dorsolateral frontal cortices). Given the role of alpha oscillations in selective attention and maintenance of working memory representations (e.g., Jensen and Mazaheri, 2010; Kelly et al., 2006) these findings suggest that many of the behavioral impairments noted in the literature may be a result of a compensatory neural response during acute hypoxia exposure. However, to date, no study has specifically examined alpha oscillations in a task-based approach specifically tied to working memory and attention. Moreover, it is also conceivable that increased alpha power and behavioral impairment are both a compensatory byproduct of some other physiological response, for example maintaining blood flow to more vital brain regions. Unfortunately, this has not been studied due to the lack of available technologies to measure brain blood flow non-invasively. Finally, the hemodynamics effects in the brain during acute hypoxia are beyond the scope of this current review.

In addition to the three main classes of data or approaches to analysis that we have described, much progress has been made in more sophisticated techniques that do not have an underlying assumption of linearity. For example, non-linear dynamic analysis has been quite popular in the larger EEG literature for the past two decades (for a review see Stam, 2005). To date, only one study has used this approach with EEG data collected under acute hypoxic conditions and found that ApEn decreased with hypoxia (Papadelis et al., 2007). While non-linear approaches provide novel insight, there is a significant issue with how to interpret the functional meaning of these results. Additional relevant information about the neural mechanisms of hypoxia may be gleaned with refinement of these non-linear approaches, exploration of connectivity analysis, and/or the use of machine learning.

### 5.1 Future directions

Here we have provided an exhaustive review of the current literature surrounding the use of EEG to evaluate the effects of hypoxia on a multitude of sensory and cognitive processes. Taken together, EEG has been shown to be a reliable and robust technological approach to developing neural markers of acute hypoxia in laboratory settings. However, there is an urgent need for the continuation and development of this work for future operational use, including (1) the feasibility of implementing EEG in operational settings to monitor air crew safety in real-time, (2) understanding the structural and metabolic correlates of acute hypoxia exposure on the human brain as they relate to current EEG findings, and (3) employing longitudinal study designs to examine what, if any, long-term consequences exist in response to repeated hypoxia exposures.

To address the first future directions, two promising efforts are currently underway to develop wearable devices that can reliably detect and inform aircrew when they have experienced a hypoxic episode. The first is an EEG device that can be worn inside the ear, similar to the foam ear plugs used for hearing protection. Mikkelsen et al. (2015) have assessed such a device (termed “ear-EEG”) and have validated its reliability by testing the ear-EEG method against the traditional scalp electrode technique using common auditory paradigms including auditory onset response, MMN, auditory steady-state response and alpha power attenuation. Their results demonstrated that the ear-EEG modality yielded similar performance results as the traditional method for spectrogram-based analyses, however, the auditory MMN was challenging to monitor (Mikkelsen et al., 2015). Given that the P3 component has been repeatedly shown to be a reliable marker of hypoxia-induced cognitive deficits, future efforts should look toward validating and implementing this paradigm along with novel recording methods such as the ear-EEG.

A second alternative to the traditional wet active electrode EEG system is a dry electrode EEG system. A study by Kam et al. (2019) introduced such a system and tested the performance of the dry system against commonly used wet EEG systems (e.g., Biosemi EEG). They employed both resting- and task-based experimental paradigms, including the P3 ERP component. Their study demonstrated that this dry EEG system performed similarly against its conventional counterpart in the P3 amplitude and topography, low frequency spectral power at rest, and in classification analyses looking at multivariate patterns of activity (Kam et al., 2019). There may be future opportunities to engineer flight helmets to have a built-in dry EEG system that would allow researchers or other crew members to continuously monitor neural electrical activity during training and tactical aviation tasks.

Future research investigating the underlying structural and metabolic correlates of disrupted signal transduction under hypoxic conditions should employ a combinatorial approach that leverages other neuroimaging technology either independent of, or together with EEG. It has been previously demonstrated in animal studies that acute hypoxia initiates a cascade of neuroinflammatory processes, including activation of two important glial cell populations: microglia (Chen et al., 2018) and astrocytes (Rueda-Carrasco et al., 2021). Healthy neurons rely heavily on these cells for normal functioning and maintenance processes. In their activated forms, glial cells become perniciously reactive, and this has been correlated with the proliferation of enhanced neuronal damage (Eltzschig and Carmeliet, 2011). The human brain relies on many compensatory mechanisms to maintain a stable environment, and neuroinflammation is known to disrupt the brain's natural homeostatic state. Presently, the effects of acute hypoxia exposure on metabolic processes in the human brain are not well understood, likely owing to the fact that these phenomena are difficult to study *in vivo*, and in isolation from the added effects of barometric pressure changes such as those observed in hypobaric hypoxia.

Advances in techniques such as magnetic resonance imaging (MRI) have allowed for investigation into even the most subtle changes in structural and functional properties associated with neuroinflammatory conditions. One notable study in military aviators used T2-weighted structural MRI to investigate brain abnormalities in US Air Force U2 pilots, who operate at altitudes of ≥ 30,000 ft above sea level (McGuire et al., 2013). Their results revealed abnormalities that were characterized by diffuse white matter hyperintensities (WMH). Furthermore, pilots who exhibited higher burdens of WMH performed significantly poorer on measures of logical reasoning, memory, performance accuracy, and general cognitive proficiency (Iacono et al., 2022; McGuire et al., 2013).

Finally, there are currently very few studies that have investigated the consequences of hypoxia on neural architecture, and no studies have considered the long-term effects of repeated hypoxia exposure over the course of a warfighter's career. This presents a limiting factor in efforts to enhance aircrew safety both in short- and long-term career periods, and even extends to aircrew longevity in the time following their retirement from military service. Identifying the neurobiological contributions to hypoxia-induced cognitive impairment, and the time course of recovery from neuronal damage, will lead to new and innovative efforts to prevent the adverse effects of hypoxia on overall brain health. Longitudinal research designs are critical in accomplishing this and could help to inform future efforts looking to prevent these episodes altogether.

### 5.2 Limitations

There are a number of recognized limitations to the study of acute hypoxia in general, as well as those specific to using EEG to assess hypoxia-related impairment. One of the major limiting factors to acute hypoxia research in this realm is the variability in methods used. First, some studies reviewed here used normobaric hypoxia while others used hypobaric hypoxia. While hypobaric hypoxia is more ecologically valid for studying exposures encountered in aviation and mountaineering, it also comes with additional safety risks that need to be weighed in a research context. For example, the US Navy phased out hypobaric chambers for hypoxia familiarization training in the 2010s, whereas the US Air Force still uses hypobaric chambers for their initial training. The use of normobaric hypoxia devices like the Reduced Oxygen Breathing Device and the Flight Breathing Awareness Trainer have gained popularity with the US Navy and Air Force due to their portability, small footprint, lower cost, and increased safety. A second variability factor in hypoxia research is the altitude or equivalent altitude that is used. In the current review, there was a range of altitudes from 10,000 ft−28,000 ft. Much of that depends on whether the motivation for the study was aviation-related or ascent to altitude, such as mountain climbing. While we might expect a dose dependent relationship in increased altitude resulting in greater impairment, the only studies that have looked at this use time of useful consciousness (Gradwell, 2016) as their outcome measure. More work is needed in the assessment of neurocognitive function, using EEG and cognitive testing, while implementing multiple altitudes in the same study.

Using EEG to study hypoxia effects also has its limitations. While EEG is a powerful tool for understanding information processing on a neural level, its form factor limits its use in real-world settings. The current gold standard is still the use of an electrode cap with “wet” electrodes, which takes time to prepare on an individual's head. However, as discussed previously, a number of technological advances in dry EEG systems, as well as in-ear devices, may make that limitation less constraining. Another limitation of EEG is its lack of spatial resolution and/or the ability to precisely investigate neural activity from deeper brain regions, such as the hippocampus. Further, EEG does not tell us the underlying causal mechanism of impairment during hypoxia. While it is clear that the electrical activity in the brain is sensitive to its oxygen supply, we do not know whether that is driven by acute neuroinflammatory responses, oxygen delivery to specific regions, changes in brain blood flow, or a number of other possibilities. For this reason, it is critical to continue investigating these mechanisms using techniques like MRI, functional near infrared spectroscopy, and transcranial doppler.

### 5.3 Conclusions

In summary, the current review has demonstrated how the use of EEG to detect neurocognitive changes that result from acute hypoxia exposure has evolved over the past several decades. Specifically, in the 2020s there has been a huge interest and investment made to advance both the acquisition, analysis, and form factor of EEG data to be more operationally relevant for use in extreme environments, such as military operations, in-flight, and mountain climbing. While true integration into operational environments will require additional development and advancement, EEG appears to have great potential for real-time physiological monitoring, especially as it relates to hypoxia-related cognitive impairment.
